# Individual and household risk factors for COVID-19 infection among household members of COVID-19 patients in home-based care in western Uganda, 2020

**DOI:** 10.1016/j.ijregi.2022.11.002

**Published:** 2022-11-11

**Authors:** Geofrey Amanya, Peter Elyanu, Richard Migisha, Daniel Kadobera, Alex Riolexus Ario, Julie R. Harris

**Affiliations:** 1 Uganda Public Health Fellowship Program, Infectious Diseases Institute; 2 Baylor College of Medicine, Kampala, Uganda; 3 National Institute of Public Health, Ministry of Health, Kampala, Uganda; 4 Division of Global Health Protection, US Centers for Disease Control and Prevention, Kampala, Uganda

**Keywords:** Screening, COVID-19, pandemic, home-based care (HBC), household contacts, Uganda, ABHR, Alcohol Based Hand Rub, AFENET, The African Field Epidemiology Network, CDC, Centers for Diseases Control and Prevention, COVID-19, Corono Virus Disease 2019, HBC, Home Based Care, MoH, Ministry of Health, PEPFAR, President's Emergency Plan for AIDS Relief, RT-PCR, Reverse Tanscription Polymerase Chain Reaction, SARS-CoV-2, Severe Acute Respiratory Syndrome Coronavirus 2, SES, Social Economic Status, TB, Tuberculosis, HH, Household

## Abstract

**Objective:**

To investigate factors associated with COVID-19 among household members of patients in home-based care (HBC) in western Uganda.

**Methods:**

We conducted a case-control and cohort study. Cases were reverse transcriptase–polymerase chain reaction-confirmed SARS-CoV-2 diagnosed 1−30 November 2020 among persons in HBC in Kasese or Kabarole districts. We compared 78 case-households (≥1 secondary case) with 59 control-households (no secondary cases). The cohort included all case-household members. Data were captured by in-person questionnaire. We used bivariate regression to calculate odds and risk ratios.

**Results:**

Case-households were larger than control-households (mean 5.8 vs 4.3 members, *P*<0.0001). Having ≥1 household member per room (adjusted odds ratio (aOR)=4.5, 95% CI 2.0−9.9), symptom development (aOR=2.3, 95% CI 1.1−5.0), or interaction with primary case-patient (aOR=4.6, 95% CI 1.4−14.7) increased odds of case-household status. Households assessed for suitability for HBC reduced odds of case-household status (aOR=0.4, 95% CI=0.2−0.8). Interacting with a primary case-patient increased the risk of individual infection among household members (adjusted risk ratio=1.7, 95% CI 1.1−2.8).

**Conclusion:**

Household and individual factors influence secondary infection risk in HBC. Decisions about HBC should be made with these in mind.

## Highlights

• Crowding increased the risk of infection
• Minimizing Interactions between the primary case and household is key to reducing SARS-CoV-2 infection
• Coughing case-patient increased risk of infection

## Introduction

The COVID-19 pandemic has stressed healthcare systems, economies and social systems around the globe. Control of spread of the causative agent, the SARS-CoV-2 virus, requires effective contact tracing and isolation of all persons testing positive until they are no longer infectious (World Health Organization 2021). In middle- and high-income settings, isolation is often possible at home if there are separate rooms, sufficient ventilation and dedicated hygiene facilities for the primary case (Patwardhan 2020). However, many households in low-income countries, including Uganda, do not have these features.

Due to the challenges with home isolation for much of the Ugandan population, institutional isolation was mandatory for confirmed cases in the early stages of the outbreak. Isolation was initially continued until the infected person tested negative; subsequently, this changed to 10−14 days after their positive test. After the first case was identified in Uganda on March 21, 2020, the outbreak grew relatively slowly, and institutional isolation of all cases was feasible (Migisha et al. 2020). However, in August 2020, cases began increasing rapidly, and as of late October 2020, approximately 12,500 cases had been confirmed, with about half of all cases occurring since August (Uganda Ministry of Health 2020).

As cases increased, healthcare facilities became stressed, and many ran out of space. Some began charging exorbitant prices for treatment outside the reach of most patients. Patients expressed increasing reluctance to go to healthcare facilities due to the hospital costs, lack of space, stigma, and myths circulating in the community about treatment (Think Well Global 2020). As a result, towards the end of 2020, the practice for patients with COVID-19 in Uganda began shifting from hospital care to home-based care (HBC).

In Uganda, HBC is intended to reflect an integrated and flexible approach to patient care and management, with a focus on family solidarity (The African Field Epidemiology Network (AFENET) 2021). It also emphasises basic traditional care patterns in Uganda, which include family members taking responsibility for providing care for their loved ones. However, those guidelines can be challenging to follow in traditional homes in Uganda, which frequently have only a single room and poor ventilation. Critical elements of HBC, such as social distancing and wearing masks inside, are often both socially challenging and logistically impossible (Neilson 2016; Pang et al. 2003).

In October 2020, there were no formalised HBC guidelines in Uganda, yet persons were beginning to be treated at home in large numbers. In November 2020, more than 750 cases were reported in the districts of Kasese and Kabarole, many of which were reported among the household members of case-patients in HBC. We sought to identify household-specific and individual factors associated with COVID-19 infection among household members of COVID-19 case-patients in the Kasese and Kabarole districts.

## Methods

### Study setting

The study was carried out in the communities of Kasese and Kabarole districts in southwestern Uganda. Fort Portal town in Kabarole District was highly affected and was the focus of the study in this district. Kasese District is located east of the Democratic Republic of Congo and west of Kabarole District. The population of Kasese District is approximately 750,000, and the population of Fort Portal town in Kabarole District is approximately 55,000 (Uganda Bureau of Statistics 2020).

### Study design

We conducted a case-control and cohort study from case-households in December 2020. To be eligible, households had to have at least 1 case-patient with confirmed SARS-CoV-2 infection being cared for in HBC settings for COVID-19. The person with the first confirmed infection in each household was defined as the primary case. At the time of this study, guidelines for HBC in Uganda were still being drafted. Despite this, some households were assessed by district health teams for their suitability before persons were enrolled in HBC. Although the criteria for selecting the households for assessment were not standardised, households were generally considered suitable for HBC if they had a dedicated separate room for the confirmed case(s); however, the decision about whether or not to place a patient in HBC was also made depending on the severity of illness and the absence of other underlying medical conditions. Only cases with asymptomatic, mild or moderate disease were considered for HBC.

#### Case-control study

The case-control study was designed to identify factors associated with infection among household members of persons in HBC. These factors included household structure, crowding (defined as a ratio of ≥1 household member per household room), ventilation (defined as ≥1 window per bedroom), and primary case characteristics. Cases were persons with reverse transcription polymerase chain reaction (RT-PCR)-confirmed infection from 1 to 30 November 2020 in Kasese District or Kabarole town. Case-households were defined as those in which at least 1 other household member beyond the primary case was infected, while control-households were those in which no other household members became infected.

#### Cohort study

Using the case-households from the above-described study, we conducted a retrospective cohort study to identify individual risk factors for contracting SARS-CoV-2 infection among all household members of primary COVID-19 patients in HBC. We collected data about each household member using a questionnaire administered either to the household member or their guardian. Data gathered included demographic factors; use of protective measures such as alcohol-based hand rub; having and using face masks and handwashing stations; comorbidities; interactions with the primary case; and knowledge about caring for the primary case. We compared exposures and outcomes among household members of case-patients with identify risk factors associated with infection.

#### Inclusion and exclusion criteria

**Figure 1:**
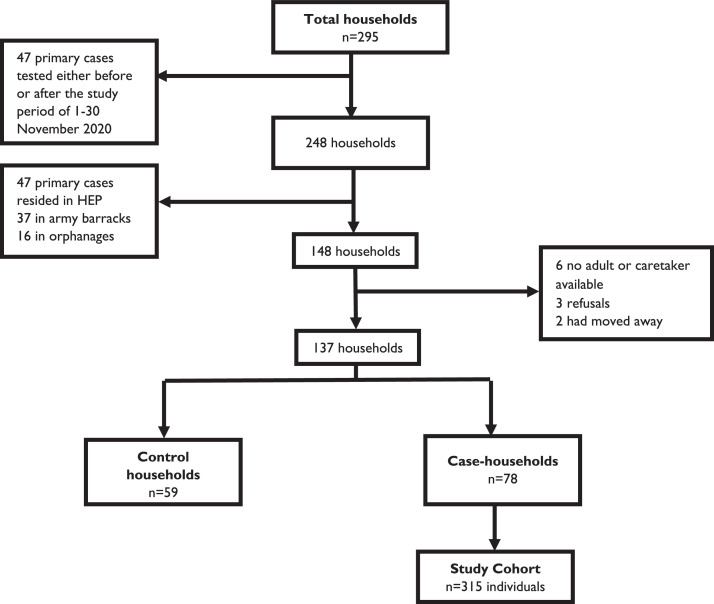
Inclusion of households and individuals in the case-control and cohort study. HEP: hydroelectric power community (closed community within Kasese district).

All homes with at least 1 person with RT-PCR-confirmed SARS-CoV-2 infection, diagnosed on 1−30 November 2020 and being treated in HBC in Kasese and Kabarole districts, were included in this study. Primary cases were tested for SARS-CoV-2 by RT-PCR either because they were symptomatic or because they were contacts of cases outside their households. Almost all household members of the primary cases were tested for SARS-CoV-2 by RT-PCR following the primary case's positive test; however, there was no protocol for this testing, and the timing of the test after the primary case's positive test varied to some extent. We excluded army barracks, orphanages, or live-at-work factories from our study due to the likelihood of their having different approaches to HBC. Households with no caretakers available, those that had relocated, and those where the head of household did not consent were excluded (Figure 1).

### Data collection and analysis

For both the case-control and the cohort study, we collected data by administering standardised questionnaires to surviving case-patients and their family members. We collected data on household construction materials, number of rooms (bedrooms and other rooms), number of household members, bedrooms and windows in a household, and the presence of electricity. We defined ‘adequate ventilation’ as a household with at least 1 window per room. We collected data from primary cases on demographic and clinical factors, including age, sex, symptoms, comorbidities and clinical outcomes. We collected exposure data, including whether a household was assessed for HBC suitability before the primary case-patient was placed in HBC, whether the case isolated or received information on how to isolate, whether the primary case had a single dedicated caretaker at home, interpersonal interactions and shared materials at home, the presence of a dedicated toilet or handwashing facility for the primary case, and the availability of face masks or alcohol-based hand rub. The facemasks that were being used at the time were double-layered cloth masks, which had been issued by the Ugandan Ministry of Health. The efficacy of these cloth masks has been described previously (Mboowa et al. 2021). In households where the primary case had died, an adult family member was interviewed as a proxy.

In the case-control study, we used bivariate analysis to explore the association of potential risk factors with being a case-household. Exposures with *P* values of <0.20 were included in multivariable models. For the multivariable model, the likelihood ratio test was used to determine if inclusion of additional covariates improved the fit of the model. Odds ratios and their associated 95% CIs were used as measures of effect size. For the comparison of case- and control-households, we calculated crude and adjusted odds ratios. For the comparison of primary cases in case- and control-households and for the cohort study, we used bivariate regression to fit univariate and multivariate generalised linear mixed effects models to identify a risk factor for secondary infections in households caring for COVID-19 patients. We reported risk ratios as our measures of association. We included variables as categorical fixed effects nested within fixed household identifiers to account for clustering. We assumed normal distribution of the random effects. Data analyses were performed using Stata software version 15 (Stata Corp, College Station, Texas).

### Ethical considerations

This activity was reviewed by the Centers for Disease Control and Prevention (CDC) and was conducted consistent with applicable federal law and CDC policy.

## Results

During November 2020, a total of 295 households with at least 1 member with laboratory-confirmed SARS-CoV-2 infection were reported to the Ministry of Health from the Kasese District. Among these, 137 were eligible for inclusion. Seventy-eight (57%) were case-households, and 59 (43%) were control-households (Figure 1).

### Case-control study

About 2/3 of both case-households and control-households were located in urban settings (towns or cities, as opposed to villages). Case-households had more household members on average than control-households (5.8 vs 4.3; *P*<0.001) (Table 1). They also had fewer rooms than control-households (mean 3.6 vs 4.5; *P*=0.003).

**Table 1:** Comparison of case- and control-household characteristics in COVID-19 HBC investigation in Kasese and Kabarole Districts, Uganda, November 2020*

Characteristic	Case HH n=78		Control HH N=59				P Value			P Value
	n	(%)	n	(%)	Unadjusted OR	95% CI		Adjusted OR	95% CI	
**Location**										
Urban	54	(69.2)	39	(66.1)	Ref					
Rural	24	(30.8)	20	(33.9)	0.9	(0.4-1.8)	0.698			
**Household members** (mean, SD)	5.8± 1.68	4.3± 2.4	—			—				
**Household member**										
≤4	17	(21.8)	39	(66.1)	Ref					
5+	61	(78.2)	20	(33.9)	7.0	(3.3-15.0)	0.000			
**Household wall material**										
Mud	6	(7.7)	14	(23.7)	Ref					
Brick	72	(92.3)	45	(77.3)	3.7	(1.3-10.4)	0.012	3.2	(1.1-9.7)	**0.037**
**Household floor material**										
Mud	13	(16.7)	12	(20.3)	Ref					
Concrete	65	(83.3)	47	(79.6)	1.3	(0.5-3.0)	0.582			
**Household roof material**										
Plant material	4	(5.1)	4	(6.8)	Ref					
Tiles / iron sheets	74	(94.9)	55	(93.2)	1.3	(0.3-5.6)	0.684			
**Rooms in HH (mean, SD)**	3.6± 1.7	4.5± 3.3								
**Rooms in HH**										
3+	13	(16.7)	29	(49.1)	Ref					
≤2	65	(83.3)	30	(50.9)	4.8	(2.2-10.6)	0.000			
**Bedrooms in HH (mean, SD)**	2.4 ± 1.1	3.1± 1.7								
**Bedrooms in HH**										
≤3	63	(80.7)	40	(67.8)	Ref					
4+	15	(19.2)	19	(32.2)	0.5	(0.2-1.1)	0.084			
**Crowding index (HH members/rooms)**										
<1 HH member / room	15	(19.2)	30	(50.9)	Ref					
≥1 HH member / room	63	(80.8)	29	(49.1)	4.3	(2.0-9.3)	0.000	4.5	(2.0-9.9)	**0.000**
**Presence of trash bin in home**										
No	36	(46.1)	33	(55.9)	Ref					
Yes	42	(53.9)	26	(44.1)	1.4	(0.8-2.3)	0.258			
**HH assessed for suitability for HBC**										
No	34	(43.6)	12	(20.3)	Ref					
Yes	44	(56.4)	47	(79.7)	0.3	(0.2-0.7)	0.005	0.4	(0.2-0.8)	**0.017**

HH: household

⁎ Other characteristics assessed that did not have an association with outcome included the presence of ≥1 window in each bedroom, having a dedicated jerrycan for the home, having electricity, having a dedicated piped water source for the home. The variables for household members and number of rooms in household were excluded from multivariable model due to collinearity with crowding index variable

### Household factors associated with case-household status

Among case-households, the mean number of infected contacts was 4.1 (median 4; range: 2–8). Crowding increased the odds of having a secondary case at home (adjusted odds ratio (aOR)=4.5; 95% CI 2.0−9.9). Having had the home assessed for suitability for COVID-19 HBC reduced the odds of case-household status (aOR=0.4, 95% CI 0.2−0.8). Having brick walls increased the odds of case-household status threefold (aOR=3.2, 95% CI 1.1−9.7). No differences were found between case- and control-households in terms of location, ventilation, having dedicated drinking water containers or piped water for the primary case, or the presence of electricity or trash bins at home (Table 1).

### Primary case factors associated with case-household status

The age of the primary case-patient was similar in control- vs case-households (41± 14.4 vs 37± 11.9 years). Having a primary case with cough (OR=7.3, 95% CI 2.7−20.0) or fever (OR=3.0, 95% CI 1.2−7.4) increased the odds of case-household status (Table 2). Having a primary case-patient who interacted with a household member increased the odds of having a secondary case (OR=6.6, 95% CI 2.5−17.8). Specifically, having someone bringing food/water (OR=4.7, 95% CI 1.9−11.6), removing dishes (OR=2.3, 95% CI 1.1−4.7), washing clothes (OR=2.6, 95% CI 1.3−5.5), or sitting with the primary case-patient inside the room (OR=3.6, 95% CI 1.5−8.6) increased the odds of having a secondary case at home. On multivariate analysis, being symptomatic (aOR=2.3, 95% CI 1.1−5.0) and interaction with the primary case (aOR=4.6, 95% CI 1.4−14.7) increased the odds of having a secondary case at home.

**Table 2:** Characteristics of primary case-patients in COVID-19 HBC investigation in Kasese and Kabarole Districts, Uganda, November 2020*

Characteristic	Case HH n=78 n (%)		Control HH N=59 n (%)		Unadjusted OR	95% CI	P Value	Adjusted OR	95% CI	P Value
**Age (mean, SD)**	37± 11.9	41± 14.4	—							
**Age**										
<20	10	(12.8)	4	(6.8)	Ref					
20-39	43	(55.1)	24	(42.4)	0.7	(0.2-2.4)	0.504	0.5	(0.1-2.3)	0.361
40+	25	(32.1)	32	(54.2)	0.3	(0.1-1.2)	0.102	0.2	(0.1-1.1)	0.076
**Male sex**	44	(56.4)	32	(54.2)	1.1	(0.6-2.2)	0.800			
**Symptoms**										
Asymptomatic	27	(34.6)	36	(61.0)	Ref					
Symptomatic (cough, fever, or difficulty breathing)*	**51**	**(65.4)**	**23**	**(39.0)**	**3.0**	**(1.5-6.0)**	**0.002**	**2.3**	**(1.1-5.0)**	**0.031**
Cough (vs asymptomatic)	33	(55.0)	6	(14.3)	7.3	(2.7-20)	0.000			
Fever (vs asymptomatic)	24	(47.1)	10	(22.7)	3.0	(1.2-7.4)	0.015			
Difficulty breathing (vs asymptomatic)	6	(18.2)	5	(12.8)	1.5	(0.4-5.5)	0.535			
**Number of HH caretakers while ill**										
Had multiple caretakers while ill	28	(35.9)	33	(55.9)	Ref					
Had single dedicated caretaker while ill	50	(64.1)	26	(44.1)	2.3	(1.1-4.5)	0.020	1.2	(0.6-3.0)	0.675
**Interactions with HH members while ill**										
None	6	(7.7)	21	(35.6)	Ref					
Any interaction (includes any of the below)*	**72**	**(92.3)**	**38**	**(64.4)**	**6.6**	**(2.5-17.8)**	**0.000**	**4.6**	**(1.4-14.7)**	**0.070**
**Brought food/water**	68	(89.5)	38	(64.4)	4.7	(1.9-11.6)	0.000			
**Took away dishes/cups**	55	(70.5)	30	(50.9)	2.3	(1.1-4.7)	0.020			
Changed bedding	26	(33.3)	13	(22.0)	1.8	(0.8-3.8)	0.149			
Washed clothes	37	(47.4)	15	(25.4)	2.6	(1.3-5.5)	0.009			
Sat with / played / talked inside room	28	(35.9)	8	(13.6)	3.6	(1.5-8.6)	0.004			
**Direct contact with HH members while ill**†										
None	23	(29.5)	21	(35.6)	Ref					
Any direct contact	55	(70.5)	38	(64.4)	1.3	(0.6-2.7)	0.449			
**Isolation at home during illness**‡										
Not isolated	20	(25.6)	10	(16.9)	Ref					
Isolated	58	(74.4)	49	(83.1)	0.6	(0.3-1.4)	0.295			
**Dedicated facilities for patient during illness**§										
No dedicated facilities	40	(51.3)	38	(64.4)	Ref					
Any dedicated facilities	38	(48.7)	21	(35.6)	0.6	(0.3-1.2)	0.126	0.7	(0.3-1.4)	0.304
**Handwashing practices while ill**										
Several times a day	63	(80.8)	51	(86.4)	Ref					
Once a day/never	15	(19.2)	8	(13.6)	1.2	(0.8-1.9)	0.381			
**Frequency of face mask use at home while ill**										
Rarely/never	10	(12.8)	7	(11.9)	Ref					
Most or all of the time	68	(87.2)	52	(88.1)	0.9	(0.3-2.6)	0.867			

HH: household

⁎ Some variables like Brought food/water, took away dishes, washed clothes, sat/played/talked inside rooms were excluded at multivariable analysis due to collinearity with any interaction, also excluded at multivariable analysis were cough and fever due to collinearity with symptoms.

† Included sharing a bed with other household members, sleeping in the same room as other household members, or using the same mobile phone as other household member. Individual variables were collinear and were therefore grouped together in analysis.

‡ Isolated at home was defined as not being in the same room as other household members while ill

§ Dedicated facilities included dedicated toilet, handwashing station/sink, and dishware/silverware

## Household member exposure factors for covid-19

### Cohort characteristics

In the 78 case-households, there were 315 household members (excluding the primary cases). Among these, 296 (94%) received a RT-PCR test for COVID-19. Among those tested, 184 (62.2%) tested positive (Table 3). Among the 296 cohort members, the median age was 21 (range: 1–71) years. Mean days between the primary case's sample collection and the cohort member's sample collection was 5.7 (median: 4, range 0−19) (Table 3).

**Table 3:** Cohort member characteristics (n=315 HH members) in COVID-19 HBC investigation in Kasese and Kabarole Districts, Uganda, November 2020.

Characteristic	n	(%)
**Sex**		
Male	105	(33.1)
Female	210	(66.7)
**Age group**		
Age (median) (range)	21(1-71)	
<5	22	(7.0)
5-11	59	(18.7)
12-19	75	(23.8)
20-39	117	(37.2)
40+	42	(13.3)
**Relationship with primary case**		
Daughter/son	102	(32.4)
Husband/wife	58	(18.4)
Sibling	53	(16.8)
Maid	45	(14.3)
Mother/father	30	(9.5)
Other relative	27	(8.6)
**Had underlying disease**	62	(19.7)
Diabetes	8	(2.5)
Hypertension	15	(4.8)
HIV	0	(0.0)
Heart disease	12	(3.8)
Lung disease	7	(2.2)
**Became symptomatic**	177	(56.2)
**Tested for COVID-19**	296	(94.0)
Tested positive for COVID-19	184	(62.2)
Tested negative for COVID-19	112	(37.8)
**Mean (median) (range) days between primary case-patient sample collection and HH member sample collection**	5.7 (4.0), (0-19)	

HH: household

Household members ≥12 years of age had an elevated risk of infection compared with those <5 years, and infection risk increased modestly with the age of the household member (Table 4). When compared with the child of the primary case, all other household members were at increased risk of infection. Household members with hypertension (all of whom were >40 years of age) were at increased risk of infection (risk ratio (RR)=1.7, 95% CI 1.6−1.9) compared with those with no comorbidities. Compared with household members who did not report interacting with the primary case, those self-reporting any form of interaction with the primary case had an increased risk for infection (RR=2.0, 95% CI 1.4−2.9). Not knowing how to care for the primary case (by self-report) was associated with increased risk of infection (RR=1.3, 95% CI 1.0−1.5), while access to alcohol-based hand rub (RR=0.7, 95% CI 0.5−0.8) or masks (RR=0.7, 95% CI 0.6−0.8) was associated with a reduced risk of infection (Table 4). Investigation of the individual impact of the interventions suggested that a combination of having a face mask plus alcohol-based hand rub or a handwashing station was protective at an individual level, compared with having a face mask alone, while the lack of a face mask was associated with increased risk (Supplementary Table 1). In the multivariate model, those that reported having any interaction were associated with increased risk of secondary infection in these households (adjusted RR=1.7, 95% CI 1.1−2.8).

**Table 4:** Risk factors for secondary COVID-19 infection among cohort members (among 296 tested) in COVID-19 HBC investigation in Kasese and Kabarole Districts, Uganda, November 2020.

Variable	Tested	Positive (N, %)		Unadjusted RR	95% CI	P value	Adjusted OR	95% CI	P value
Sex									
Female	101	61	(60.4)	Ref					
Male	195	124	(62.6)	0.9	(0.7-1.3)	0.782	-	-	-
Age group									
<5	16	8	(50.0)	Ref					
5-11	55	34	(61.8)	2.0	(0.7-5.6)	0.208	2.1	(0.6-11.0)	0.212
12-19	71	52	(72.3)	2.5	(0.9-7.0)	0.075	2.4	(0.5-10.8)	0.251
20-39	112	77	(68.8)	2.6	(0.9-7.1)	0.062	1.9	(0.4-8.9)	0.395
40+	42	35	(85.7)	3.3	(1.2-9.4)	0.023	2.2	(0.5-10.8)	0.311
Relationship to primary case									
Daughter/son	96	40	(41.7)	Ref					
**Husband/wife**	**56**	**47**	**(83.9)**	**2.0**	**(1.3-3.1)**	**0.000**	**1.6**	(0.9-3.0)	0.110
Sibling	47	29	(61.7)	1.5	(0.9-2.3)	0.107	1.4	(0.8-2.4)	0.194
Maid	42	28	(66.7)	1.6	(0.9-2.6)	0.056	1.5	(0.9-2.7)	0.156
Mother/father	29	20	(68.9)	1.7	(0.9-2.8)	0.066	1.5	(0.8-2.8)	0.160
**Other relative**	**26**	**20**	**(76.9)**	**1.8**	**(1.1-3.2)**	**0.025**	**1.7**	(0.8-3.1)	0.123
Comorbidities									
None	235	137	(58.3)	Ref					
Any	61	47	(77.1)	1.3	(0.9-1.9)	0.099	1.0	(0.6-1.4)	0.988
Has hypertension	15	15	(100.0)	1.7	(0.9-2.8)	0.059	1.4	(0.7-2.7)	0.326
Has diabetes	8	4	(50.0)	0.9	(0.2-2.1)	0.659	-	-	-
Has heart disease	12	8	(66.7)	1.1	(0.5-1.6)	0.840	-	-	-
Has lung disease	7	4	(57.1)	0.9	(0.3-2.5)	0.865	-	-	-
Interaction with primary case									
None	57	20	(35.1)	Ref					
**Any interaction**	**239**	**164**	**(68.6)**	**2.0**	**(1.2-3.1)**	**0.005**	**1.7**	**(1.1-2.8)**	**0.028**
Mask access									
No	76	56	(71.1)	Ref					
Yes	220	128	(59.1)	0.6	(0.4-0.9)	0.011	3.8	(0.4-19.6)	0.205
Glove access									
No	279	173	(62.0)	Ref					
Yes	17	6	(35.3)	0.9	(0.5-1.7)	0.891	-	-	-
Alcohol-based hand rub access									
No	222	154	(83.7)	Ref					
**Yes**	**74**	**30**	**(16.3)**	**0.6**	**(0.4-0.9)**	**0.007**	0.2	(0.1-1.4)	0.102
Handwashing station at home									
No	61	42	(68.9)	Ref					
Yes	235	142	(60.4)	0.9	(0.6-1.2)	0.457		-	-
Knowledge about how to care for COVID-19 patient at home									
Knew how	154	87	(47.3)	Ref					
Did not know how	142	97	(54.9)	1.3	(0.9-1.7)	0.130	1.3	(0.9-1.8)	0.115

HH: household. RR: risk ratio

## Discussion

We identified multiple factors associated with infection among household members of COVID-19 patients in HBC in Uganda. These findings have important implications in Uganda, where safe HBC is critical to stemming the epidemic. Crowding at home and having a coughing or febrile primary case-patient were associated with increased risk of infection among household members, as were interactions with the primary case. Specific household members were also at increased risk, with increasing age significantly associated with infection. Access to some protective measures, but not others, was associated with lower risk of individual infection, as was knowledge (self-reported) about how to care for the primary case.

Increasing household size was associated with increased risk for infection, which is to be expected as additional household members increase the opportunity for onward transmission. To account for this, we used a crowding metric to evaluate household risk and found that crowding at home increased the odds of having secondary cases more than four-fold. Crowding has previously been associated with both increased transmission and severity of respiratory infections (Cevik et al. 2020), possibly related to limited space in a household, fewer opportunities for ventilation and/or longer or more direct exposure to index cases (Leclerc et al. 2020; Villela 2021). This reflects a particular challenge in Uganda, where the average household size is 4.6 persons, yet 45% of households have only one room for sleeping (Uganda Bureau of Statistics 2019; Okonkwo et al. 2020). Uganda's guidelines for HBC recommend that a separate room be made available for a patient in HBC but do not address alternate approaches if there is crowding (Uganda Ministry of Health 2021). As an alternative, they suggest a separation of 2 meters between shared spaces, which might not always be possible, especially in rural settings in Uganda.

We also identified that having brick walls, compared with mud walls, was an independent risk factor for case-household status. This finding was somewhat unexpected, as having brick walls is considered to be associated with a higher household socioeconomic status than having mud walls, and lower socioeconomic status has been shown to be associated with both COVID-19 (Foster et al. 2022) and influenza risk (Mamelund, Shelley-Egan, and Rogeberg 2021) in other settings. In Uganda, an association between higher-quality housing (as measured by having brick versus mud walls) and risk of household COVID-19 transmission may have been driven by other unmeasured factors, including ventilation of the households. For example, brick-walled households could have been more likely to have closed glass windows, compared with mud-walled households, which might be more likely to have open-air windows. Natural ventilation has been shown to reduce transmission of other airborne diseases, such as tuberculosis (Melissa Lygizos 2013; Adrian Roderick Escombe 2007). We measured ventilation only by the presence or absence of windows rather than the window material or actual ventilation. Measurement of actual ventilation in future studies might inform the underlying reasons for this difference in risk.

Although spreading of COVID-19 from asymptomatic persons to contacts has been clearly demonstrated (Wilmes et al. 2021; Luo et al. 2020), it is thought to be associated with a lower secondary attack rate when compared with spread from symptomatic individuals (Li et al. 2021). In our study, having a coughing or febrile primary case was independently associated with increased odds of subsequent infection in the household. The HBC guidelines for Uganda recommend the use of control measures such as mask-wearing and social distancing for all household members to reduce transmission risk (Uganda Ministry of Health 2021). However, we noted that mask access for primary cases–typically considered a protective intervention against both aerosol- and droplet-transmitted infections (Sterr et al. 2021; Kähler and Hain 2020)−did not protect household members, while mask access by other household members was protective for them. In addition, interactions between the primary case and household members increased risk for household members and access to alcohol-based hand rub was protective. These results suggest that fomite or droplet spread may play a role in household transmission of COVID-19. However, it may also relate to inadequate mask materials, poor mask fit, or poor adherence to mask use by the primary case, which we did not measure in this study. Interestingly, direct contact with the primary case did not increase risk. However, this may reflect that bedroom-sharing in Uganda often includes the entire family rather than just the spouse (Uganda Bureau of Statistics 2019). In homes with other risk factors for infection among household members, it may be worth considering the symptomatology of the primary case when making decisions about the appropriateness of HBC. In addition, simple and inexpensive interventions such as alcohol-based hand rub and sufficient masks for the duration of infectiousness of the primary case could be considered as part of a package distributed to or recommended to HBC households.

At the time of this study, assessment of households for suitability for HBC before enrolment in HBC was irregular, enabling us to study this variable as a risk factor for secondary infections in households caring for COVID-19 patients. Both having the household assessed for suitability for HBC and knowledge (self-reported) about how to care for the primary case at home were protective. Currently, multiple materials exist to guide persons on appropriate HBC (Uganda Ministry of Health 2021; Centers for Disease Control and Prevention 2020). During periods when HBC care is occurring, these could be disseminated broadly in Uganda in ways that are appropriate for the setting, such as newspaper, radio and television, through community leaders, and in local languages.

This study had some limitations. First, we were unable to conclusively confirm which person was the true primary case in case-households. While many household members were tested after the primary case, in some cases, the testing was done on the same day as the primary case. In these situations, we relied on the first report of symptoms to identify the likely primary case. However, we could not rule out another household member being the true primary case or alternately a common exposure between some case-patients who were household members. While this could have confounded some of our analyses of individual risk factors, it is unlikely to impact the assessment of household risk factors. Second, it is possible household members were exposed in multiple ways, such as from ongoing exposure to community transmission; therefore, the primary case might not have been the source of infection among other household members. Unfortunately, we did not ask about other potential sources of infection for household members and were unable to assess this. Third, some misclassification may have taken place among control-households; it is possible that secondary cases occurred but were not identified. This would lead to an overestimation of odds ratios for the association between household factors and illness. Fourth, some members of case-households could have developed infection after the first round of testing of household members, resulting in misclassification of some infected persons as uninfected, which could have overestimated odds ratios for individual risk factors for illness. Fifth, we interviewed proxies in households where the primary case-patient had died, which could have introduced recall bias, although this bias is not likely to be differential across case- and control-households. Finally, it is possible there might have been recall bias among case-households due to the multiple cases in a single home, leading contacts to recall more carefully their interactions with the primary case than in control-households. This would have the likely effect of increasing the apparent association between exposure and outcome.

### Recommendations

Special considerations should be applied to HBC in Uganda, given the local setting and practices. For example, small crowded homes, those in which a household member has underlying disease, or situations in which the primary case is symptomatic or actively coughing may not be appropriate for HBC. Homes should be assessed for suitability and provided with or encouraged to purchase hand sanitiser and masks, and interactions between the primary case- and household members should be minimised. A clear, multilingual manual for homes engaging in HBC on how to care for persons with COVID-19 may also be useful.

## Appendix Supplementary materials

Image, application 1
