# Supplementary material for: Individual and household risk factors for COVID-19 infection among household members of COVID-19 patients in home-based care in western Uganda, 2020
Source: IJID Reg. 2022 Nov 11;5:183–90. doi: 10.1016/j.ijregi.2022.11.002 (PMC9650255; doi:10.1016/j.ijregi.2022.11.002)
Supplement: Supplementary file 1 [file mmc1.docx]

**Supplementary Table 1**

Risk of testing positive, comparing persons with different combinations of non-pharmaceutical interventions to those having only a mask, in COVID-19 HBC investigation in Kasese and Kabarole Districts, Uganda, November 2020.

| **Mask** | **Gloves** | **ABHR** | **Handwashing station** | **Tested** | **Positive** | **RR** | **95% CI** |
| --- | --- | --- | --- | --- | --- | --- | --- |
| - | - | - | - | 5 | 5 | 1.3 | (1.1-1.5) |
| + | - | - | - | 33 | 26 | REF |  |
| - | + | - | - | 0 | 0 | -- | -- |
| - | - | + | - | 13 | 9 | 0.87 | (0.59-1.3) |
| - | - | - | + | 15 | 15 | 1.3 | (1.1-1.5) |
| + | + | - | - | 3 | 2 | 0.85 | (0.37-1.9) |
| + | - | + | - | 6 | 0 | 0.092 | (0.0063-1.3) |
| + | - | - | + | 153 | 97 | 0.80 | (0.65-1.0) |
| - | + | + | - | 1 | 0 | 0.32 | (0.029-3.6) |
| - | + | - | + | 1 | 1 | 0.63 | (0.16-2.6) |
| - | - | + | + | 35 | 26 | 0.94 | (0.72-1.2) |
| + | + | + | - | 0 | 0 | -- |  |
| + | - | + | + | 19 | 0 | 0.032 | (0.0021-0.50) |
| + | + | - | + | 10 | 3 | 0.38 | (0.015-1.0) |
| - | + | + | + | 2 | 0 | 0.21 | (0.017-2.7) |
| + | + | + | + | 0 | 0 | -- |  |
| **TOTAL** | | | | **296** | **184** |  |  |
